# Analysis of the initial lot of the CDC 2019-Novel Coronavirus (2019-nCoV) real-time RT-PCR diagnostic panel

**DOI:** 10.1371/journal.pone.0260487

**Published:** 2021-12-15

**Authors:** Justin S. Lee, Jason M. Goldstein, Jonathan L. Moon, Owen Herzegh, Dennis A. Bagarozzi, M. Steven Oberste, Heather Hughes, Kanwar Bedi, Dorothie Gerard, Brenique Cameron, Christopher Benton, Asiya Chida, Ausaf Ahmad, David J. Petway, Xiaoling Tang, Nicky Sulaiman, Dawit Teklu, Dhwani Batra, Dakota Howard, Mili Sheth, Wendi Kuhnert, Stephanie R. Bialek, Christina L. Hutson, Jan Pohl, Darin S. Carroll

**Affiliations:** 1 Division of Scientific Resources, Centers for Disease Control and Prevention, National Center for Emerging Zoonotic Infectious Diseases, Atlanta, Georgia, United States of America; 2 Division of Viral Diseases, Centers for Disease Control and Prevention, National Center for Infectious Respiratory Diseases, Atlanta, Georgia, United States of America; 3 Centers for Disease Control and Prevention, Office of the Deputy Director for Infectious Diseases, Atlanta, Georgia, United States of America; 4 Division of High Consequence Pathogens and Pathology, Centers for Disease Control and Prevention, National Center for Emerging Zoonotic Infectious Diseases, Atlanta, Georgia, United States of America; University of Helsinki: Helsingin Yliopisto, FINLAND

## Abstract

At the start of the COVID-19 pandemic, the Centers for Disease Control and Prevention (CDC) designed, manufactured, and distributed the CDC 2019-Novel Coronavirus (2019-nCoV) Real-Time RT-PCR Diagnostic Panel for SARS-CoV-2 detection. The diagnostic panel targeted three viral nucleocapsid gene loci (N1, N2, and N3 primers and probes) to maximize sensitivity and to provide redundancy for virus detection if mutations occurred. After the first distribution of the diagnostic panel, state public health laboratories reported fluorescent signal in the absence of viral template (false-positive reactivity) for the N3 component and to a lesser extent for N1. This report describes the findings of an internal investigation conducted by the CDC to identify the cause(s) of the N1 and N3 false-positive reactivity. For N1, results demonstrate that contamination with a synthetic template, that occurred while the “bulk” manufactured materials were located in a research lab for quality assessment, was the cause of false reactivity in the first lot. Base pairing between the 3’ end of the N3 probe and the 3’ end of the N3 reverse primer led to amplification of duplex and larger molecules resulting in false reactivity in the N3 assay component. We conclude that flaws in both assay design and handling of the “bulk” material, caused the problems with the first lot of the 2019-nCoV Real-Time RT-PCR Diagnostic Panel. In addition, within this study, we found that the age of the examined diagnostic panel reagents increases the frequency of false positive results for N3. We discuss these findings in the context of improvements to quality control, quality assurance, and assay validation practices that have since been improved at the CDC.

## Introduction

The complete SARS-CoV-2 genome sequence from a patient in Wuhan, China, was published on January 12, 2020 (NCBI: NC_045512.2). Shortly thereafter, CDC began development of the CDC 2019-nCoV Real-Time RT-PCR Diagnostic Panel (referred to hereafter as the ‘diagnostic panel’). There was an urgent need to design, validate, manufacture, and distribute a diagnostic assay rapidly. The original diagnostic panel design targeted three nucleocapsid (*N)* gene loci, each with its own specific primers and probe [1]. N is a structural protein and a target for both antigen-based diagnostic assays and antiviral drug development [2]. N1 and N2 primers and probes are specific to the *N* gene of SARS-CoV-2. The N3 components were designed for detection of a conserved region of the *N* gene from SARS-CoV-2 and other closely related coronaviruses (S1 Fig). The rationale for this third component was to ensure detection of SARS-CoV-2 even if mutations occurred in other regions of the *N* gene [3, 4]. The instructions for interpretation of results were “*When all controls exhibits the expected performance and the cycle threshold growth curve for any one or two markers (N1*, *N2*, *N3) (but not all three markers) crosses the threshold line within 40*.*00 cycles (<40*.*00 Ct) the result is inconclusive*. *Repeat extraction and rRT-PCR*. *If the repeated results remains inconclusive*, *contact CDC immediately for instructions for transfer of specimen to CDC for additional testing and further guidance*.” The diagnostic panel contained additional materials including: (i) a process control comprising primers and a probe that targeted the human *RNase P* gene to demonstrate proper specimen collection; (ii) a human specimen control (HSC) and associated primers/probe to demonstrate successful extraction and integrity of the extracted nucleic acid; and (iii) a positive control (target containing N1, N2, N3, and RP primer and probe binding sites).

Early manufacturing processes were performed in three distinct sites within the CDC’s laboratory facilities. Synthesis, purification, and chemical analysis of primer and probe oligonucleotides were performed by the CDC Lab 1. Preparation of the bulk material (combining individual syntheses of primers and probes to produce a large volume) and initial quality analysis were performed by CDC Lab 2. Dispensing of the primers and probes into individual vials, drying, labeling, and kit assembly were performed by CDC Lab 3.

The distribution of diagnostic panel kits to state public health laboratories began on February 5, 2020 for use under a Food and Drug Administration (FDA) Emergency Use Authorization (EUA) [1]. Within several days, CDC received reports from multiple laboratories of fluorescence in the absence of viral template (false-positive reactivity) for both the N1 and N3 oligonucleotide sets. The false-positive reactivity for N1 was not observed in subsequent production lots but the false reactivity in the N3 oligonucleotide set persisted and led to the eventual removal of the N3 primers and probes from the diagnostic panel assay.

The most likely underlying causes of RT-PCR fluorescence in the absence of target (viral nucleic acids or positive control material) include contamination of reagents with homologous template molecules or an assay design flaw that results in cleavage of the quencher and dye from the probe during replication by a polymerase [5]. To investigate the cause(s) of the false-positive reactivity in the original N1 and N3 assay components, we evaluated the performance and products from the diagnostic panel using three different sources of oligonucleotides: (i) Reference validation reagents (pre-EUA; an aliquot of the materials from the internal validation of the assay, with primers and probes manufactured at CDC to EUA specifications prior to the first lot of EUA reagent manufacturing); (ii) EUA kit components manufactured at CDC (EUA-kit; Lot #20–0121 –a diagnostic panel kit from the original lot that was distributed to public health labs); and (iii) components produced by an external, commercial vendor.

The diagnostic panel assay was replicated according to the reaction conditions described in the FDA EUA Instructions For Use for all three sources of reagents. For each set of oligonucleotides, one 96-well plate of RT-PCR reactions was run that included positive controls (the assay positive control included the exact sequence of the SARS-CoV-2 Wuhan-Hu-1 sequence for each target region (S1 Fig)) and no-template controls (NTC—containing water but no nucleic acid template). To characterize the molecules amplified during the positive control reactions and those with false-positive reactivity, representative post-reaction products were evaluated by capillary electrophoresis (to estimate product sizes) and Illumina sequencing (Illumina Inc., San Diego, CA).

## Methods

This work involved analyses of the performance of this assay in the absence of any human samples, thus no human subjects were involved in this work.

### Generation of RT-PCR products

RT-PCR was performed using the ABI 7500 Fast Dx Real-Time PCR instrument (Applied Biosystems, Foster City, CA) in Standard 7500 Run Mode. For the EUA-kit oligonucleotides (~1 month from the lot initiation of Lot# 20–0121), one 96-well plate of RT-PCR reactions was run that included four positive control wells (E12, F12, G12, H12), four human specimen control wells (A12, B12, C12, D12), and 88 no-template control wells (Columns 1 through 11). Additionally, for the EUA-kit oligonucleotides (~21 months from the lot initiation of Lot# 20–0121) 4 more 96-well plates of RT-PCR reactions were run which included 3 positive control wells and 93 no-template control wells. For the pre-EUA and commercial vendor oligonucleotides, one 96-well plate of RT-PCR reactions was run that included three positive control wells (F12, G12, H12) and 93 no template control wells. All RT-PCR reactions were performed using the same reaction components and thermal cycling conditions. The master mix consisted of 8.5 μL nuclease-free water, 1.5 μL primer/probe mix, 5.0 μL TaqPath^™^ 1-Step RT-qPCR Master Mix (4x) per reaction. Five μL of water or control nucleic acid was added to the designated wells for a final reaction volume of 20 μL. The following thermal cycling conditions were used: Stage 1, 2 minutes at 25°C; Stage 2, 15 minutes at 50°C; Stage 3, 2 minutes at 95°C; Stage 4, 45 cycles; Stage 4, Step 1, 3 seconds at 95°C; Stage 4, Step 2, 30 seconds at 55°C.

### Capillary electrophoresis

The dsDNA 905 reagent kit (1–500 bp) was used to analyze RT-PCR amplicons by a Fragment Analyzer 5200 (Agilent, Santa Clara, CA). Briefly, 2 μL of each RT-PCR amplicon was mixed with 22 μL diluent buffer 1 x TE in sample plate. The marker plate was set up and the ready-to-use ladder was added to the corresponding well according to manufacturer’s instruction. PROSize 3.0 software was used for the data analysis. Relative fluorescent markers consist of Lower Marker (LM) and Upper Marker (UM) and used as calibrators to determine oligonucleotide average size (within range low-high detected base pairs) and concentration in sample calculated by RFU (relative fluorescent units) and corresponding peak area. Total Integrated Concentration (TIC) is the concentration of all detected peaks, Total Integrated Molarity (TIM) is the molarity of all detected peaks, both excluding LM and UM. According to the documentation for the instrument and reagent kit, the coefficient of variation is < 10% for automated definition of estimated peak size. We analyzed pooled samples (7 individual reactions) from EUA Kits of Lot# 20–0121 consisting of N1 NTC reactions or N3 NTC reactions, as well as two N1 pre-reaction primers and probe samples. Further analysis consisted of 12 individual samples from two EUA Kits of Lot# 20–0121 representing N3 NTC RT-PCR products, as well as 2 individual samples from commercial N3 NTC RT-PCR products.

### Characterization of RT-PCR products through next-generation sequencing

Sequencing libraries were prepared from unpurified RT-PCR products using the NEBNext Ultra DNA Library Preparation Kit (New England Biolabs Inc., Ipswich, MA) modified for very short insert sizes [6]. The libraries were pooled at approximately equimolar ratios and sequenced using the Illumina MiSeq Micro v2 kit (300 cycles) (Illumina Inc., San Diego, CA). Demultiplexed FastQ files were generated using bcl2fastq v2.20 with default settings but adding a setting to preserve short-trimmed reads. Adapters and low-quality bases were trimmed and merged using FastP v0.20.1 with default parameters but again removing the limit for minimum read length [7]. Merged reads were mapped to the SARS-CoV2 Wuhan-Hu-1 (NCBI: MN908947) N gene target sequences in Geneious Prime 2019 using high sensitivity settings, modified to include a minimum mapping quality of 20 and a maximum gap size of 2 bp [8]. Unmapped reads were then mapped to all nine N1, N2, and N3 primer and probe sequences using the same parameters. All alignments were visually inspected to verify accuracy.

### *In silico* reagent analysis

OligoAnalyzer software (Integrated DNA Technologies, Coralville, IA) was used to calculate the propensity of primer and probe sequences for duplex and hairpin formation. Thermodynamic ΔG was calculated for Hetero- and Homo-dimer secondary structures and reported as a problematic design if ΔG ≤-9 kcal/mol.

## Results

### Analysis of N1 primers and probes

Real-time RT-PCR assays were performed on the pre-EUA, EUA-kit, of N1 diagnostic panel oligonucleotides. False reactivity of the N1 oligonucleotides in NTC wells was observed only with the EUA-kit components, but not with the pre-EUA primers and probes (Table 1). The EUA-kit N1 NTC reaction with false reactivity evaluated by capillary electrophoresis demonstrated multiple distinct populations of DNA molecules (S2 Fig). The highest concentration was for unreacted primers and probes at 20 bases in length (S2 and S3 Figs), followed by lower concentrations at approximately 42 and 55 bases in length (primer hetero-duplexes) (S4 Fig). There was no detectable evidence of a 72 base pair (bp) amplicon indicative of target amplification or template contamination.

**Table 1:** Summary of RT-PCR and sequencing results from no-template control reactions with multiple reagent production sources of the CDC 2019-Novel Coronavirus (2019-nCoV) real-time reverse transcriptase RT-PCR diagnostic panel.

Reagent source	N1 target	N3 target
Reference Validation Reagents (pre-EUA)	0% false positive	0.5–2% false positive
		Ct values 33–38
		Sequence: Primers and probe interaction
Emergency Use Authorization material (EUA-kit)	2% false positive	97% false positive
	Ct values 38	Ct values 34–39
	Full length product	Sequence: Primers and probe interaction
	Sequence: Contaminant DNA	
Commercial Vendor	NT	0.5–2% false positive
		Ct values 34–39
		Sequence: Primers and probe interaction

NT—not tested in this evaluation. The N2 components were never reported to result in false reactivity and therefore were not part of this evaluation.

**Fig 1:**
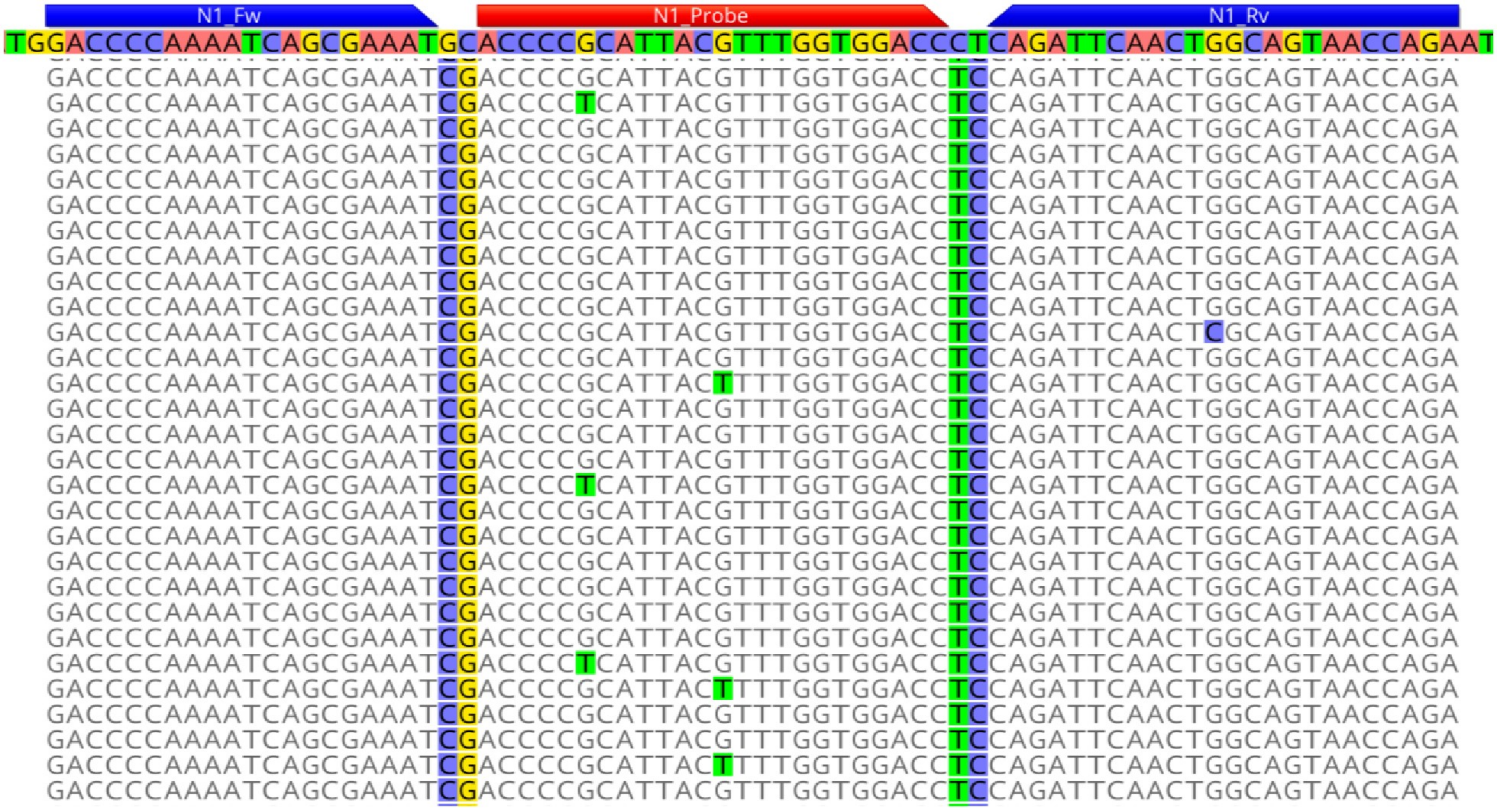
Next generation sequence data from products of the EUA Kit N1. 34% of reads generated from the EUA-kit N1 NTC reaction with false-positive reactivity mapped to the SARS-CoV-2 Wuhan-Hu-1 reference sequence (highlighted sequence at top) but contained four distinguishing SNPs (highlighted in the mapped reads) consistent with a contaminating template synthesized at CDC around the same time as the kit production (S1a Fig). The N1 primer and probe binding sites are annotated above the reference sequence.

Upon sequencing of the PCR products from the EUA-kit N1 reaction with false reactivity, the majority of NGS reads (inserts post-adapter and quality trimming) were similar in length to the putative duplexes observed by capillary electrophoresis (S5 Fig—size estimates differ slightly between capillary electrophoresis and NGS output). Importantly, however, among the NGS output there was a third population of molecules at 72 base pairs in length. When the NGS reads were mapped to the SARS-CoV-2 Wuhan-Hu-1 reference sequence, approximately 34% of reads from the EUA-kit N1 product with false reactivity mapped to the reference sequence at the specific target site of the N1 primers and probes (Fig 1). All of these reads contained four single nucleotide polymorphisms (SNPs) that are identical to a synthetic oligonucleotide template produced at CDC around the time of EUA kit production. The SNPs clearly distinguish the template as not originating from SARS-CoV-2 nucleic acids or other components of the diagnostic panel such as the positive control (S1a Fig). The remainder of the reads comprised homo- and hetero-duplex molecules involving the N1 forward and N1 reverse primers (S4 Fig). Less than 1% of the oligonucleotide reads involved the probe sequence (Table 2) and therefore, other than the contaminating template, no other sources of fluorescence were identified in the sequencing data.

**Table 2:** Sequencing results of RT-PCR products demonstrated the source of false reactivity in N1 and N3 components.

RT-PCR Components^i^	Reagent Source^ii^	% Reads Mapped to Reference^iii^	% Template Contaminant^iv^	% Reads Mapped to Oligonucleotides	% Reads Involving Probe^v^
N1_pc (n = 1)	EUA-kit	96%	nd	4%	<1%
N1_fp (n = 2)	EUA-kit	nd	34% (0%)	66% (0%)	<1% (0%)
N3_fp (n = 2)	pre-EUA	nd	nd	98% (1%)	51% (2%)
N3_pc (n = 1)	EUA-kit	42%	nd	58%	<1%
N3_fp (n = 14)	EUA-kit	nd	nd	>99% (0%)	37% (4%)
N3_fp (n = 6)	Commercial	nd	nd	94% (6%)	43% (10%)

nd: not detected.

^i)^ pc: positive control; fp: false-positive reactivity during RT-PCR.

^ii)^ EUA-kit: Emergency Use Authorization (first lot of CDC distributed kits); pre-EUA: an aliquot of the materials from the internal CLIA validation of the assay; Commercial: components ordered from a commercial supplier.

^iii)^ reference is the CDC 2019-Novel Coronavirus Real-Time RT-PCR Diagnostic Panel positive control which is derived from the Wuhan-Hu-1 sequence (GenBank accession number MN908947).

^iv)^ synthetic template produced by CDC at approximately the same time as kit production containing differentiating bases (Fig 1).

^v)^ the percent of merged reads that contained partial or complete probe sequences and therefore could contribute to false reactive signal during RT-PCR. Due to library preparation, the proportion of NGS reads in each category may not accurately reflect the proportion of product in the RT-PCR output.

**Fig 2:**
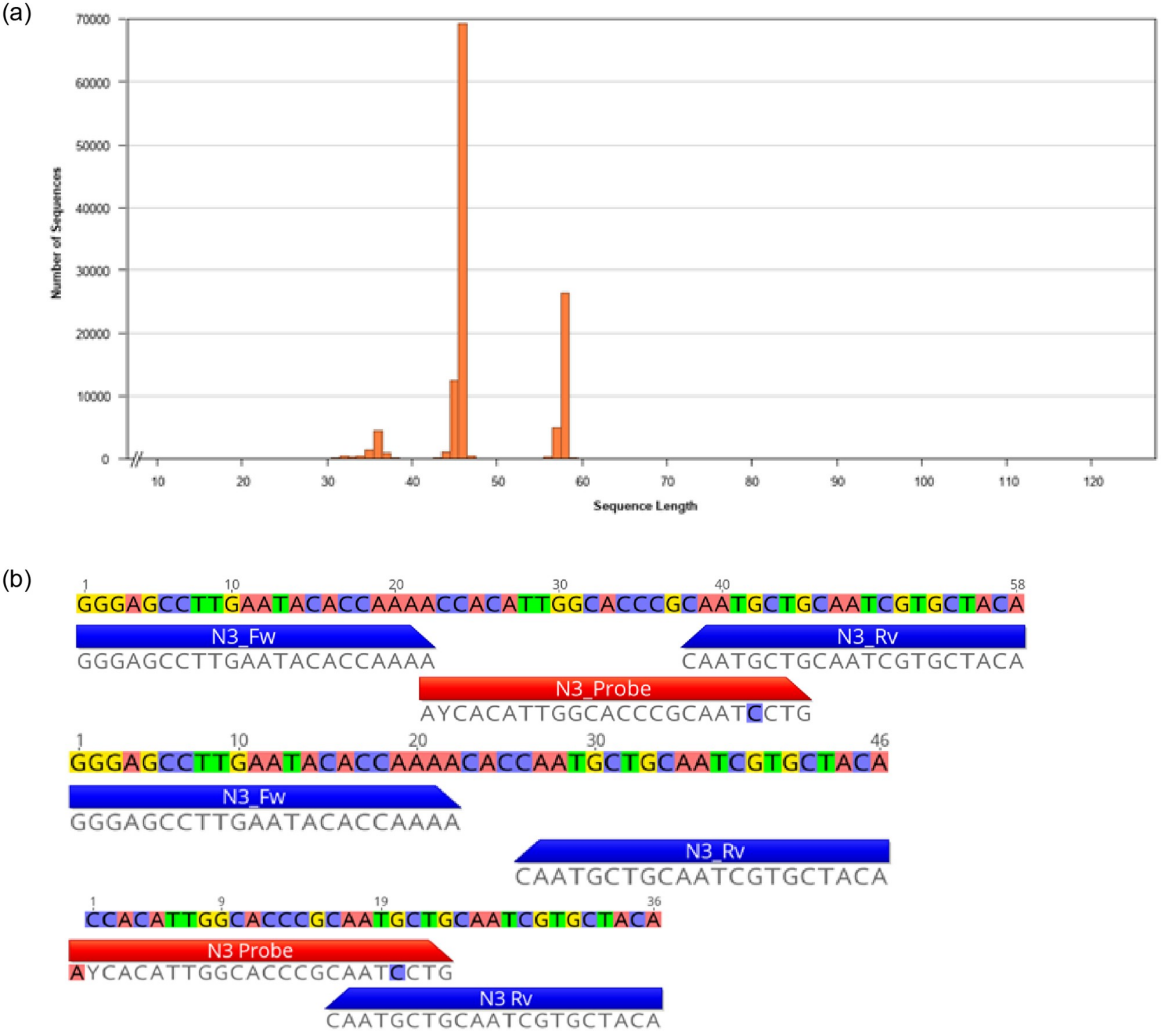
a. The NGS read length distribution (insert sizes after adapter and quality trimming) from the EUA-kit N3 false positive product demonstrating three prominent populations of molecules at 36 bp, 46 bp, and 58 bp in length. All three populations were identified as originating from the N3 assay components and comprise oligonucleotide duplex or triplex molecules (Fig 2b). b. Representative NGS reads from the three most common populations of the EUA-kit N3 false-positive RT-PCR reaction products (Fig 2a). N3 primers and probes are mapped below each read (reverse primer shown in reverse-complement). The 58 bp product and 36 bp products involve the probe and therefore would have generated fluorescence in the absence of template during the reaction. These same populations of products were generated from all three sets of primers and probes (pre-EUA, EUA-kit, commercial vendor).

The first lot of CDC N1 EUA components was contaminated with a synthetic template (see also Fig 1; S1a and S1b Fig). The N3 components form multimeric molecules involving the probe, leading to fluorescence in the absence of template (Fig 2b). The latter was consistent regardless of the source of oligonucleotides tested. Mean values with standard deviation in parentheses shown for all results with n > 1.

Based on these findings, and given that the false reactivity of the N1 primers and probes was not seen in subsequent lots of EUA oligonucleotides produced at the CDC, we conclude that the source of N1 false-positive reactivity in the first lot of the EUA diagnostic panel released to public health laboratories was due to contamination of the kits by a synthetic oligonucleotide. Because the EUA kits were contaminated, but not the pre-validation material (pre-EUA), the contamination must have occurred during the post production quality control process or packaging of the EUA kits distributed to public health labs.

### Analysis of N3 primers and probes

All three sets of N3 oligonucleotides tested (pre-EUA, EUA-kit, and commercial vendor) produced some false-positive reactivity of the N3 primers and probes in the NTC wells from RT-PCR (Table 1). The proportion of NTC reactions with false reactivity was highest in the EUA-kit materials. The size distribution of EUA-kit N3 RT-PCR products as visualized by capillary electrophoresis indicated the presence of unreacted primers (peak around 16 bases), and oligonucleotide duplex molecules (peaks around 55 and 65 bases in length) (S6 Fig). There was no observed peak at 72 bp that would be consistent with template contamination. Similar size distributions were present in RT-PCR products from the pre-EUA and commercial vendor oligonucleotides.

The majority of NGS reads generated from the products of the EUA-kit N3 reaction with false reactivity were approximately 46 bp or 58 bp in length, with a smaller population at approximately 36 bp (Fig 2a). There were no reads with insert lengths of 72 bp matching the sequence of the reference or positive control. None of the reads mapped cleanly to the target location of the SARS-CoV-2 Wuhan-Hu-1 reference sequence between the N3 primer binding sites (S7 Fig). When the NGS reads from the N3 NTC RT-PCR products were mapped to the diagnostic panel primers and probes, between 29% and 61% of the products were duplex or triplex involving the N3 probe, and thus would have generated fluorescence during the RT-PCR reaction in the absence of template (Table 2; Fig 2b—36 bp and 58 bp reads). This pattern was similar regardless of the source of oligonucleotides evaluated (pre-EUA, EUA-kit, and commercial vendor). Consistent with these NGS results, *in silico* analysis of the diagnostic panel assay components predicts multiple regions of complementarity between the 3’ end of the N3 probe and the 3’ end of the N3 reverse primer (S2 and S8 Figs). The remainder of the reads comprised oligonucleotide duplex molecules involving the N3 forward and N3 reverse primers only (Fig 2b—46 bp read). These duplexes were also common in the products from the N3 positive control (Table 2; S5c Fig).

Without any evidence of contamination, and with consistent amplification of N3 primer-probe complexes across three different sources of oligonucleotides, we conclude that the false-positive reactivity among the N3 components of the diagnostic assay was due to an assay design flaw. Complementarity between the N3 probe and N3 reverse primer resulted in amplification of duplex and triplex molecules, emitting fluorescence during the RT-PCR assay in the absence of target or other template molecules. Given that the rate of false reactivity varied from low (0.5–2%) to high (100%) depending on the source of the oligonucleotides (Table 1), the frequency of the false reactivity appears to be highly variable and at this time we do not know what factors may influence the outcome.

## Discussion

The N1 and N3 components of the first distribution (Lot #20–0121) of the CDC 2019-Novel Coronavirus (2019-nCoV) Real-Time RT-PCR Diagnostic Panel suffered from sporadic false-positive reactivity. This issue was resolved for N1 with subsequent production lots, but the problem persisted for N3 and those components were eventually removed from the EUA assay. The evaluation described in this report determined the source of the fluorescence in the early N1 components to be a contaminating template molecule, which was not part of the diagnostic panel assay design, but was synthesized at the CDC around the same time as the manufacturing of the first EUA lot. This template was present in Lab 2 where the “bulk” panel components underwent the original quality analyses and it is likely that the contamination of the N1 bulk material occurred at that time. No other likely source of fluorescence was identified in the sequence data from the N1 RT-PCR products. We also conclude that the design of the N3 components led to primer-probe amplification, explaining the persistence of false reactivity with these oligonucleotides across multiple EUA production lots and across the three sources of primers and probes used in this evaluation. There was no evidence of contaminating molecules or other source of fluorescence in the sequence data from the N3 RT-PCR products. The rate of false-positive reactivity varied greatly however there was a clear pattern where false reactivity increased along with the age (time since manufacture, EUA-kit oligonucleotides tested at ~1 and ~21 months from lot initiation of Lot# 20–0121) of the diagnostic panels analyzed. Given the identified likelihood of false signal due to N3 primer probe interactions, it is likely that the combination of these materials in one tube in the diagnostic panel could have increased these false signals as the panels age and are subjected to freeze thaw cycles. This also could explain why early evaluation runs, which used newly produced reagents that had not been previously combined did not see such high levels of false signal.

In response to the problems with the early performance of the diagnostic panel, CDC implemented a more comprehensive and rigorous review process during the development of the CDC Influenza SARS-CoV-2 (Flu SC2) Multiplex Assay, which detects SARS-CoV-2, influenza A virus, and influenza B virus in upper or lower respiratory tract specimens [9]. This process included review and approval of the assay concept, intended use, and design as well as evaluation and verification by experts from multiple CDC programs independent of the assay design team. The validation of the Flu SC2 assay included extensive and iterative testing involving multiple CDC staff from multiple scientific programs. Prior to finalizing the assay design, CDC also sought input on the targets and assay conditions from external assay development experts and piloted the assay with three public health laboratories to confirm its functionality and usability.

## Supporting information

S1 Fig Alignments demonstrating the N1 and N3 target molecules and the single nucleotide polymorphisms (SNPs) that distinguish the assay positive control sequences from other synthetic templates manufactured at CDC around the time of kit production. a. N1 target reference sequence (positive control; MN908947—top) and synthetic template sequence (202002553 –bottom). Primer and probe locations are annotated above the reference sequence. Distinguishing SNPs are highlighted. The synthetic template was sequenced in N1 false-positive samples (Fig 1), providing evidence that false-reactivity in the N1 RT-PCR reactions was due to contamination in the kit. b. N3 target reference sequence (positive control; MN908947—top) and synthetic template sequence (202002557—bottom). Primer and probe locations are annotated above the reference sequence. Distinguishing SNPs are highlighted. No evidence of template contamination was present in NGS data from false-positive N3 RT-PCR reactions regardless of oligonucleotide source.
(DOCX)

S2 Fig Capillary electrophoresis analysis of EUA-kit N1 NTC reaction detecting oligonucleotide peaks with median size of 20 bp (non-reacted primers and probe) and 55 bp (putative homo- and hetero-duplexes). Additional minor peaks at 42 bp and 51 bp were also present. Oligonucleotides with calculated free energy (ΔG) are predicted for low energy interaction < -3.0 kcal∙mole-1 and duplex formation <8.9 kcal∙mole-1. Maximum free energy is based on 100% complementary sequence. The number of theoretical pairs favorable for duplex formation are listed on right side of chart.
(DOCX)

S3 Fig Capillary electrophoresis analysis of EUA-kit N1 pre-reaction primers and probe detecting a primary peak at 20 bp. (DOCX)

S4 Fig Representative reads from the most common oligonucleotide (non-template) products produced from the EUA-kit N1 RT-PCR reactions with false reactivity (see S5a Fig). N1 primers are annotated below the NGS reads (highlighted bases above).
(DOCX)

S5 Fig Next-generation sequencing read-length distribution from diagnostic panel EUA-kit RT-PCR products. S5a: N1 NTC (false positive); S5b: N1 positive control; S5c: N3 positive control (N3 false positive is shown in Fig 2a). For all three panels, the x-axes are scaled to show all read lengths present and y-axes are scaled relative to abundance of reads in each sample. a. The EUA-kit N1 false positive product contained two populations of short non-specific oligonucleotide duplex molecules and a prominent peak at 72 bp. The 72 bp reads correspond to the contaminating template molecules present in the EUA N1 components (Fig 1). The shorter products at 44 bp and 52 bp in length involved hetero-duplexes of the N1 forward and N1 reverse primers (S4 Fig) and thus, other than the contaminating template, no other source of fluorescence was identified in the output of the N1 RT-PCR reaction. b. The EUA-kit N1 positive control product demonstrated expected template product at a length of 72 bp and very few shorter non-target reads. c. The EUA-kit N3 positive control product contained two primary read lengths—the expected product at 72 bp and another more abundant set of reads at 46 bp in length comprising N3-Fw and N3-Rv duplex molecules (Fig 2b).
(DOCX)

S6 Fig Capillary electrophoresis analysis of EUA-kit N3 NTC RT-PCR products. Fragment Analyzer detection of a residual primers/probes at ~15 bp, as well as putative homo- and hetero-duplex molecules at 55 bp and 65 bp.
(DOCX)

S7 Fig Alignment of EUA-kit N3 NTC reads mapped to the SARS-CoV-2 Wuhan-Hu-1 reference sequence (highlighted sequence at top). The N3 primer and probe binding sites are annotated above the reference sequence. 99% of reads were oligonucleotide duplex or triplex molecules (Table 1; Fig 2). No reads were identified of the length and sequence that would indicate the presence of contamination in the N3 components. These results were similar across all three sources of N3 primers and probes (pre-EUA, EUA-kit, commercial vendor).
(DOCX)

S8 Fig Oligo-Analyzer online software analysis for predicted duplex. Oligonucleotide pairs with most favorable free energy (ΔG < -8.9 kcal∙mole^-1^) are listed for each N1 and N3 oligonucleotide pair. **A**. N1 primer/probe ΔG prediction for heteroduplex (N1-F/N1-P) of -8.91 kcal∙mole^-1^, **B**. N3 primer/probe ΔG prediction for heteroduplex (N3-R/N3-P) of -10.09 kcal∙mole^-1^. Predicted complementary nucleotide bases that contribute to free energy are denoted with dots and lines (contiguous sequence).
(DOCX)
