# Supplementary material for: Analysis of the initial lot of the CDC 2019-Novel Coronavirus (2019-nCoV) real-time RT-PCR diagnostic panel
Source: PLoS One. 2021 Dec 15;16(12):e0260487. doi: 10.1371/journal.pone.0260487 (PMC8673615; doi:10.1371/journal.pone.0260487)
Supplement: S1 Fig — a. N1 target reference sequence (positive control; MN908947—top) and synthetic template sequence (202002553 –bottom). Primer and probe locations are annotated above the reference sequence. Distinguishing SNPs are highlighted. The synthetic template was sequenced in N1 false-positive samples (Fig 1), providing evidence that false-reactivity in the N1 RT-PCR reactions was due to contamination in the kit. b. N3 target reference sequence (positive control; MN908947—top) and synthetic template sequence (202002557—bottom). Primer and probe locations are annotated above the reference sequence. Distinguishing SNPs are highlighted. No evidence of template contamination was present in NGS data from false-positive N3 RT-PCR reactions regardless of oligonucleotide source. (DOCX) [file pone.0260487.s001.docx]

**S1 Figure. Alignments demonstrating the N1 and N3 target molecules and the single nucleotide polymorphisms (SNPs) that distinguish the assay positive control sequences from other synthetic templates manufactured at CDC around the time of kit production.**


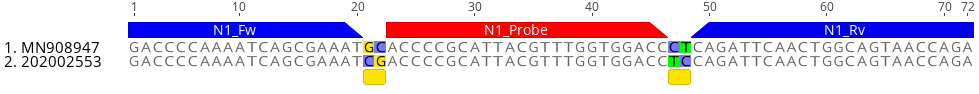


**Figure S1a. N1 target reference sequence** (positive control; MN908947 - top) **and synthetic template sequence** (202002553 – bottom). Primer and probe locations are annotated above the reference sequence. Distinguishing SNPs are highlighted. The synthetic template was sequenced in N1 false-positive samples (Figure 1), providing evidence that false-reactivity in the N1 RT-PCR reactions was due to contamination in the kit.


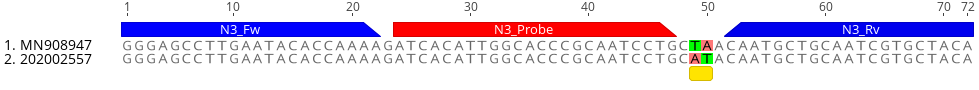


**Figure S1b**. **N3 target reference sequence** (positive control; MN908947 - top) **and synthetic template sequenc**e (202002557 - bottom). Primer and probe locations are annotated above the reference sequence. Distinguishing SNPs are highlighted. No evidence of template contamination was present in NGS data from false-positive N3 RT-PCR reactions regardless of oligonucleotide source.
