# Supplementary material for: Analysis of the initial lot of the CDC 2019-Novel Coronavirus (2019-nCoV) real-time RT-PCR diagnostic panel
Source: PLoS One. 2021 Dec 15;16(12):e0260487. doi: 10.1371/journal.pone.0260487 (PMC8673615; doi:10.1371/journal.pone.0260487)
Supplement: S2 Fig — Additional minor peaks at 42 bp and 51 bp were also present. Oligonucleotides with calculated free energy (ΔG) are predicted for low energy interaction < -3.0 kcal∙mole-1 and duplex formation <8.9 kcal∙mole-1. Maximum free energy is based on 100% complementary sequence. The number of theoretical pairs favorable for duplex formation are listed on right side of chart. (DOCX) [file pone.0260487.s002.docx]

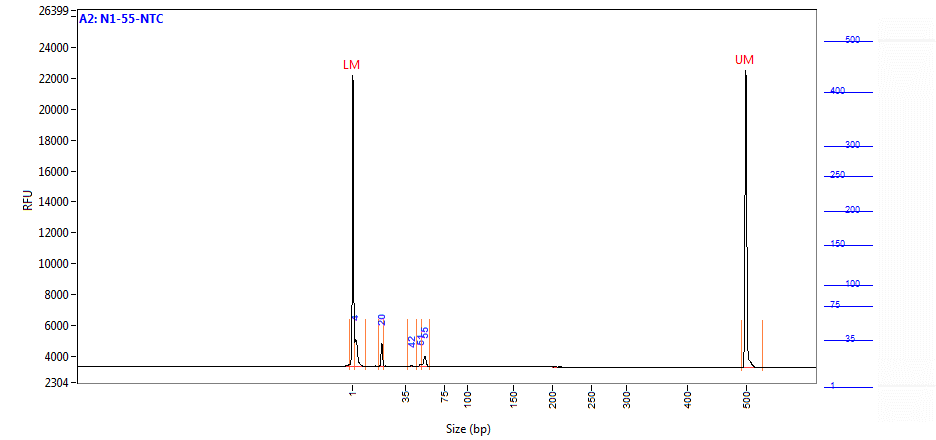


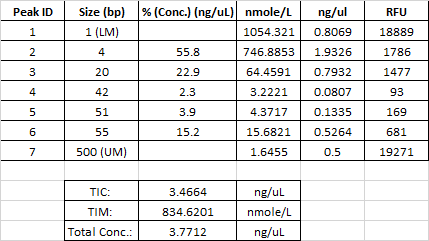


**S2 Figure. Capillary electrophoresis analysis of EUA-kit N1 NTC reaction** detecting oligonucleotide peaks with median size of 20 bp (non-reacted primers and probe) and 55 bp (putative homo- and hetero-duplexes). Additional minor peaks at 42 bp and 51 bp were also present.
