# Supplementary material for: Analysis of the initial lot of the CDC 2019-Novel Coronavirus (2019-nCoV) real-time RT-PCR diagnostic panel
Source: PLoS One. 2021 Dec 15;16(12):e0260487. doi: 10.1371/journal.pone.0260487 (PMC8673615; doi:10.1371/journal.pone.0260487)
Supplement: S3 Fig — (DOCX) [file pone.0260487.s003.docx]

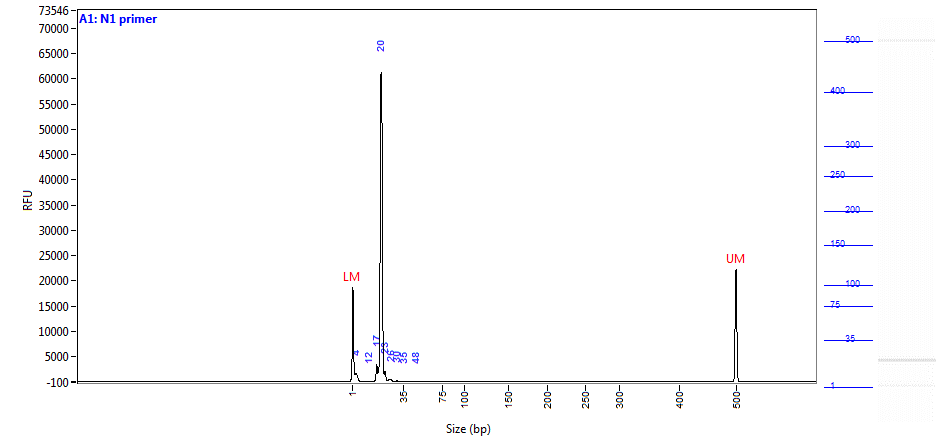


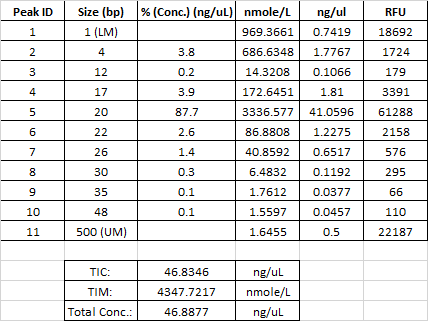


**S3 Figure. Capillary electrophoresis analysis of EUA-kit N1 pre-reaction primers and probe** detecting a primary peak at 20 bp.
