# Supplementary material for: Analysis of the initial lot of the CDC 2019-Novel Coronavirus (2019-nCoV) real-time RT-PCR diagnostic panel
Source: PLoS One. 2021 Dec 15;16(12):e0260487. doi: 10.1371/journal.pone.0260487 (PMC8673615; doi:10.1371/journal.pone.0260487)
Supplement: S4 Fig — N1 primers are annotated below the NGS reads (highlighted bases above). (DOCX) [file pone.0260487.s004.docx]

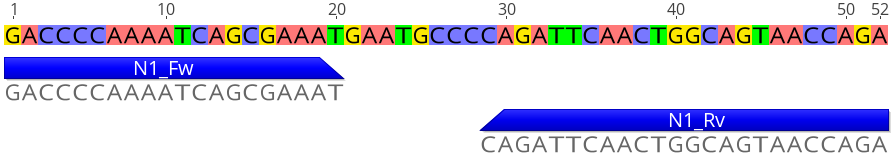


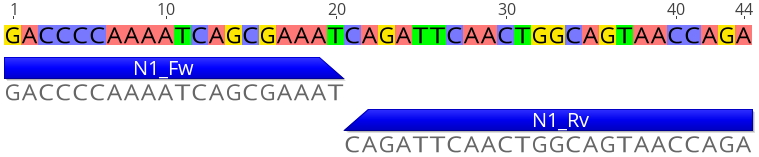


**S4 Figure**. **Representative reads from the most common oligonucleotide (non-template) products** produced from the EUA-kit N1 RT-PCR reactions with false reactivity (see Figure S5a). N1 primers are annotated below the NGS reads (highlighted bases above).
