# Supplementary material for: Analysis of the initial lot of the CDC 2019-Novel Coronavirus (2019-nCoV) real-time RT-PCR diagnostic panel
Source: PLoS One. 2021 Dec 15;16(12):e0260487. doi: 10.1371/journal.pone.0260487 (PMC8673615; doi:10.1371/journal.pone.0260487)
Supplement: S5 Fig — S5a: N1 NTC (false positive); S5b: N1 positive control; S5c: N3 positive control (N3 false positive is shown in Fig 2a). For all three panels, the x-axes are scaled to show all read lengths present and y-axes are scaled relative to abundance of reads in each sample. a. The EUA-kit N1 false positive product contained two populations of short non-specific oligonucleotide duplex molecules and a prominent peak at 72 bp. The 72 bp reads correspond to the contaminating template molecules present in the EUA N1 components (Fig 1). The shorter products at 44 bp and 52 bp in length involved hetero-duplexes of the N1 forward and N1 reverse primers (S4 Fig) and thus, other than the contaminating template, no other source of fluorescence was identified in the output of the N1 RT-PCR reaction. b. The EUA-kit N1 positive control product demonstrated expected template product at a length of 72 bp and very few shorter non-target reads. c. The EUA-kit N3 positive control product contained two primary read lengths—the expected product at 72 bp and another more abundant set of reads at 46 bp in length comprising N3-Fw and N3-Rv duplex molecules (Fig 2b). (DOCX) [file pone.0260487.s005.docx]

**S5 Figure.** **Next-generation sequencing read-length distribution from diagnostic panel EUA-kit RT-PCR products**. S5a: N1 NTC (false positive); S5b: N1 positive control; S5c: N3 positive control; N3 false positive is shown in Figure 2a). For all three panels, the x-axes are scaled to show all read lengths present and y-axes are scaled relative to abundance of reads in each sample.


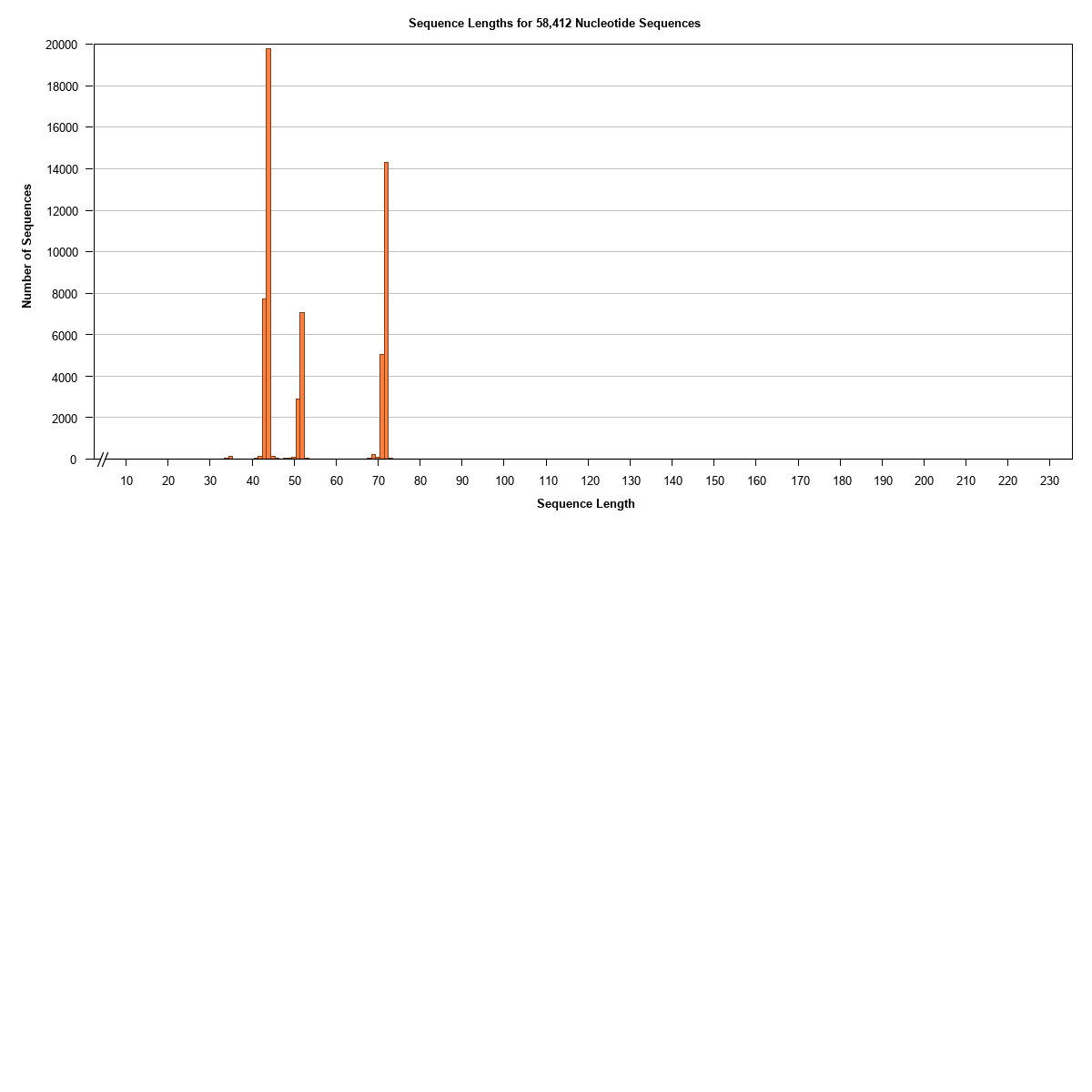


**S5a Figure**. **The EUA-kit N1 false positive product contained two populations of short non-specific oligonucleotide duplex molecules and a prominent peak at 72 bp**. The 72 bp reads correspond to the contaminating template molecules present in the EUA N1 components (Figure 1). The shorter products at 44 bp and 52 bp in length involved hetero-duplexes of the N1 forward and N1 reverse primers (Figure S4) and thus, other than the contaminating template, no other source of fluorescence was identified in the output of the N1 RT-PCR reaction.


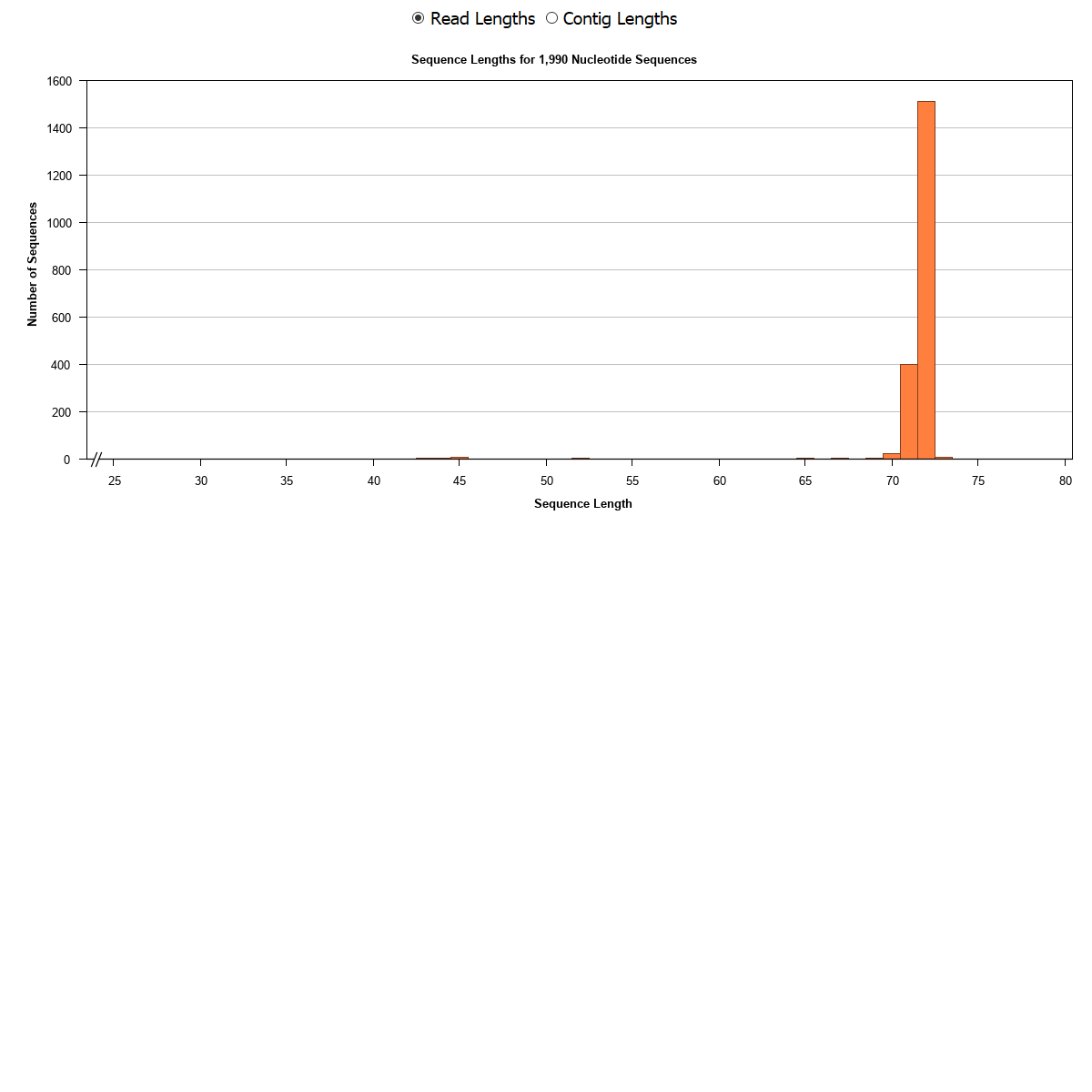


**S5b Figure.** **The EUA-kit N1 positive control product demonstrated expected template product** at a length of 72 bp and very few shorter non-target reads.


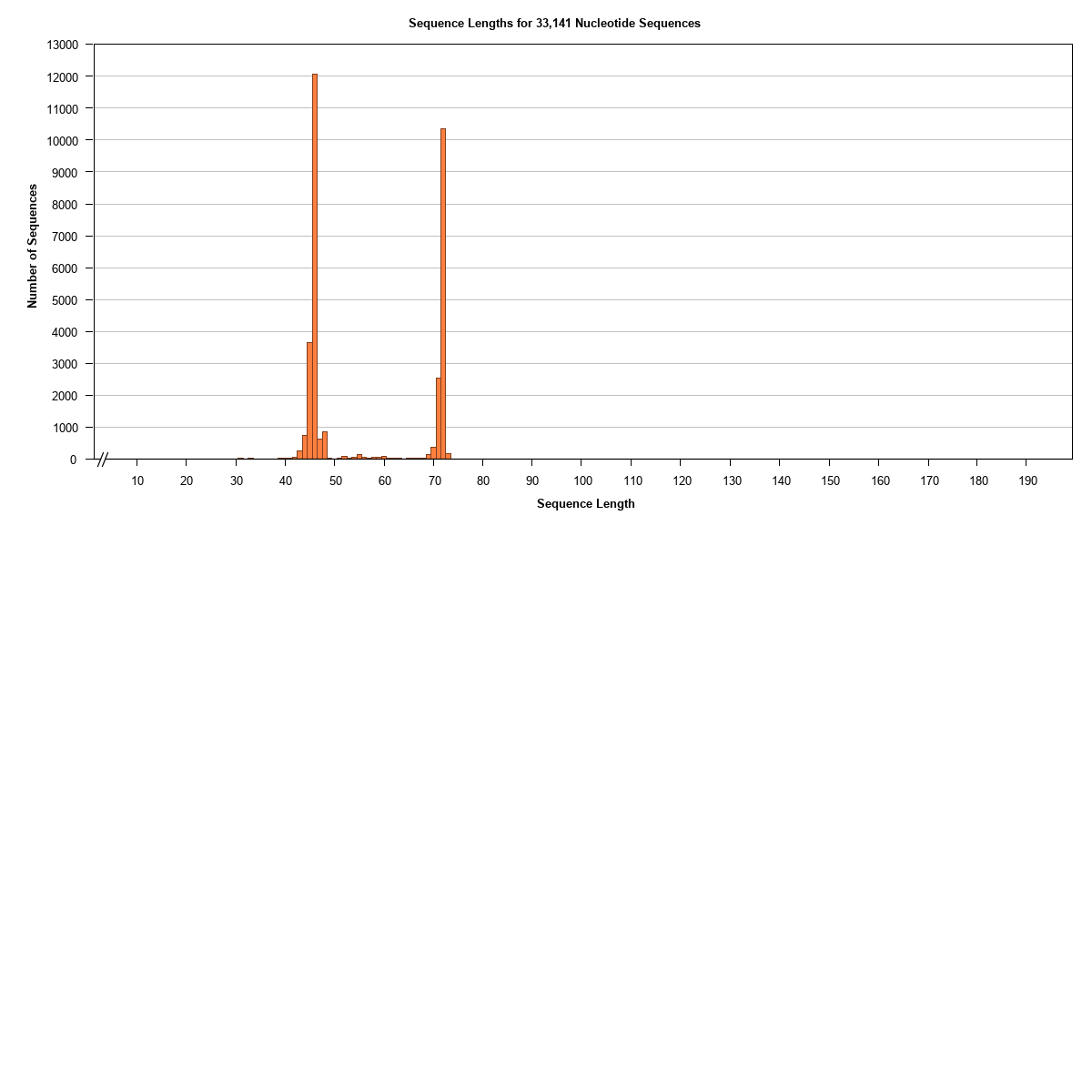


**S5c Figure.** **The EUA-kit N3 positive control product contained two primary read lengths** - the expected product at 72 bp and another more abundant set of reads at 46 bp in length comprising N3-Fw and N3-Rv duplex molecules (Figure 2b).
