# Supplementary material for: Analysis of the initial lot of the CDC 2019-Novel Coronavirus (2019-nCoV) real-time RT-PCR diagnostic panel
Source: PLoS One. 2021 Dec 15;16(12):e0260487. doi: 10.1371/journal.pone.0260487 (PMC8673615; doi:10.1371/journal.pone.0260487)
Supplement: S6 Fig — Fragment Analyzer detection of a residual primers/probes at ~15 bp, as well as putative homo- and hetero-duplex molecules at 55 bp and 65 bp. (DOCX) [file pone.0260487.s006.docx]

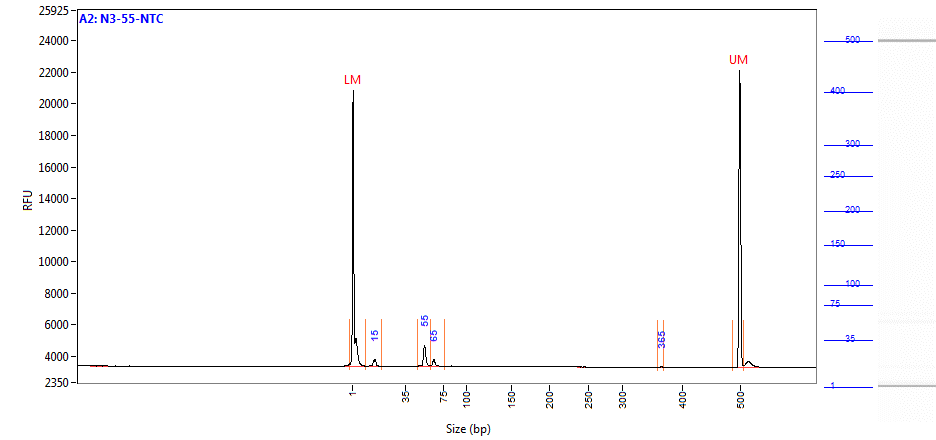


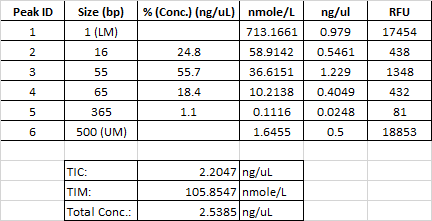


**S6 Figure. Capillary electrophoresis analysis of EUA-kit N3 NTC RT-PCR products**. Fragment Analyzer detection of a residual primers/probes at ~15 bp, as well as putative homo- and hetero-duplex molecules at 55 bp and 65 bp.
