# Supplementary material for: Analysis of the initial lot of the CDC 2019-Novel Coronavirus (2019-nCoV) real-time RT-PCR diagnostic panel
Source: PLoS One. 2021 Dec 15;16(12):e0260487. doi: 10.1371/journal.pone.0260487 (PMC8673615; doi:10.1371/journal.pone.0260487)
Supplement: S7 Fig — The N3 primer and probe binding sites are annotated above the reference sequence. 99% of reads were oligonucleotide duplex or triplex molecules (Table 1; Fig 2). No reads were identified of the length and sequence that would indicate the presence of contamination in the N3 components. These results were similar across all three sources of N3 primers and probes (pre-EUA, EUA-kit, commercial vendor). (DOCX) [file pone.0260487.s007.docx]

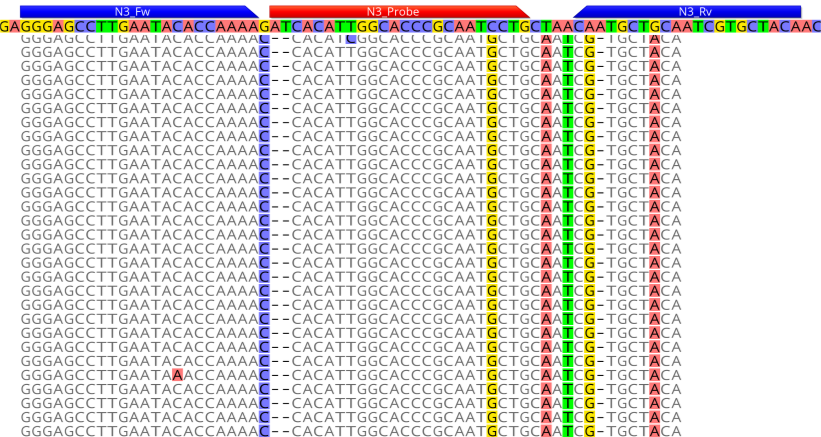


**S7 Figure.** **Alignment of EUA-kit N3 NTC reads mapped to the SARS-CoV-2 Wuhan-Hu-1 reference sequence** (highlighted sequence at top).  The N3 primer and probe binding sites are annotated above the reference sequence. 99% of reads were oligonucleotide duplex or triplex molecules (Table 1; Figure 2).  No reads were identified of the length and sequence that would indicate the presence of contamination in the N3 components.  These results were similar across all three sources of N3 primers and probes (pre-EUA, EUA-kit, IDT).
