# Supplementary material for: Analysis of the initial lot of the CDC 2019-Novel Coronavirus (2019-nCoV) real-time RT-PCR diagnostic panel
Source: PLoS One. 2021 Dec 15;16(12):e0260487. doi: 10.1371/journal.pone.0260487 (PMC8673615; doi:10.1371/journal.pone.0260487)
Supplement: S8 Fig — Oligonucleotide pairs with most favorable free energy (ΔG < -8.9 kcal∙mole-1) are listed for each N1 and N3 oligonucleotide pair. A. N1 primer/probe ΔG prediction for heteroduplex (N1-F/N1-P) of -8.91 kcal∙mole-1, B. N3 primer/probe ΔG prediction for heteroduplex (N3-R/N3-P) of -10.09 kcal∙mole-1. Predicted complementary nucleotide bases that contribute to free energy are denoted with dots and lines (contiguous sequence). (DOCX) [file pone.0260487.s008.docx]

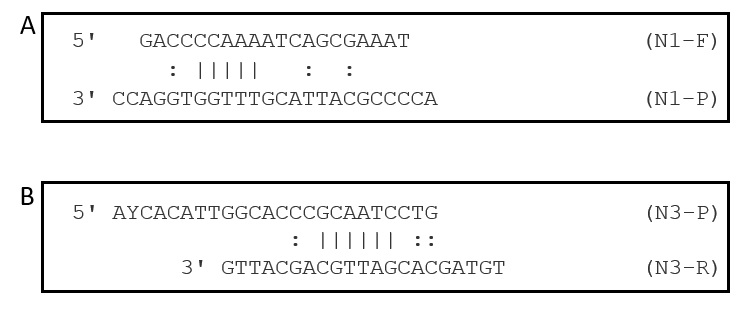


**S8 Figure. Oligo-Analyzer online software analysis for predicted duplex**. Oligonucleotide pairs with most favorable free energy (ΔG < -8.9 kcal∙mole^-1^) are listed for each N1 and N3 oligonucleotide pair. **a**. N1 primer/probe ΔG prediction for heteroduplex (N1-F/N1-P) of -8.91 kcal∙mole^-1^, **b**. N3 primer/probe ΔG prediction for heteroduplex (N3-R/N3-P) of -10.09 kcal∙mole^-1^**.** Predicted complementary nucleotide bases that contribute to free energy are denoted with dots and lines (contiguous sequence).
